# A Case Report of Neuropathic Arthropathy in Chronic Syrinx Raising Suspicion of Malignancy

**DOI:** 10.7759/cureus.61746

**Published:** 2024-06-05

**Authors:** Sushma Edara, Sivaprasad Nalluri

**Affiliations:** 1 Internal Medicine, Interfaith Medical Center, New York City, USA; 2 Internal Medicine, South Dayton Acute Care Consultants, Dayton, USA

**Keywords:** syringomyelia, charcot joint of shoulder, shoulder, neuropathic arthropathy, charcot's joint

## Abstract

Syringomyelia is a prevalent cause of Charcot arthropathy, notably affecting the elbow and less frequently the shoulder. Before attributing neuropathic arthropathy (NA) to a syrinx, careful investigation of various potential causes is vital. We present a unique case of NA affecting the left shoulder, secondary to a longstanding syrinx presenting as an expansile mass on imaging, raising suspicion of malignancy. The patient presented with progressive left arm swelling, limited mobility, and a history of chronic left shoulder pain. Through clinical evaluation and imaging, including X-rays and CT scans, significant bone destruction and a large fluid-filled mass in the left shoulder were observed. Laboratory tests ruled out other potential diagnoses, and a bone biopsy excluded malignancy. This study emphasizes the importance of thorough differential diagnosis and appropriate imaging techniques to distinguish NA from other conditions. The diagnosis of NA relies on a comprehensive assessment involving clinical signs, symptoms, radiological imaging, and additional tests aimed at excluding other potential causes, including soft tissue tumors. Management strategies, including conservative approaches and surgical interventions like neurosurgical decompression and shoulder arthroplasty, are discussed. The study sheds light on the challenges in diagnosing and managing NA associated with syringomyelia and emphasizes the significance of a multidisciplinary approach for optimal outcomes.

## Introduction

Syringomyelia, marked by a fluid-filled syrinx within the spinal cord that causes compression of the spinothalamic tract fibers, results in the dissociation of pain and temperature sensation. This neurological deficit precipitates neuropathic arthropathy (NA), a severe degenerative joint disorder, notably manifesting as Charcot arthropathy in non-weight-bearing joints like the shoulder. The case we present involves an elderly female patient with noticeable swelling and restricted mobility in her left shoulder joint. After a thorough analysis of her medical history, clinical presentation, and diverse diagnostic examinations, we contemplated several potential causes for NA affecting the shoulder. This case sheds light on the complexities of differential diagnosis and underscores the significance of exploring alternative origins before definitively diagnosing NA linked to syringomyelia.

## Case presentation

A 70-year-old female, previously diagnosed with insulin-dependent type 2 diabetes mellitus and hypertension, and who had undergone a laminectomy for cervical and thoracic syringomyelia in 2015, presented to the emergency department due to progressive left arm swelling over a five-day period. The symptoms began with swelling around the left shoulder and gradually progressed, causing heaviness and swelling in the arm, forearm, and fingers. Review of the patient's medical records revealed a history of chronic left shoulder pain with limited range of motion, notably improved after the laminectomy and physical therapy. However, the patient had lost touch with medical follow-ups since then. Interestingly, she denied experiencing any numbness or tingling sensations in her arm.

During the physical examination, an elderly, obese female was observed with normal range of motion in the cervical spine. She exhibited moderate edema in the left upper extremity, extending from the shoulder joint to the fingers when compared to the right arm. There was marked limited range of motion at the left shoulder joint but normal range of motion at the left elbow, wrist, and fingers. The patient could actively abduct her arm by 20° using accessory muscles, and passive abduction and flexion were possible up to 90°. However, she was unable to hold her arm against gravity and could only be adducted passively. No tenderness, erythema, warmth, or lymphadenopathy was noted upon palpation, and her sensation and temperature were intact.

**Figure 1:**
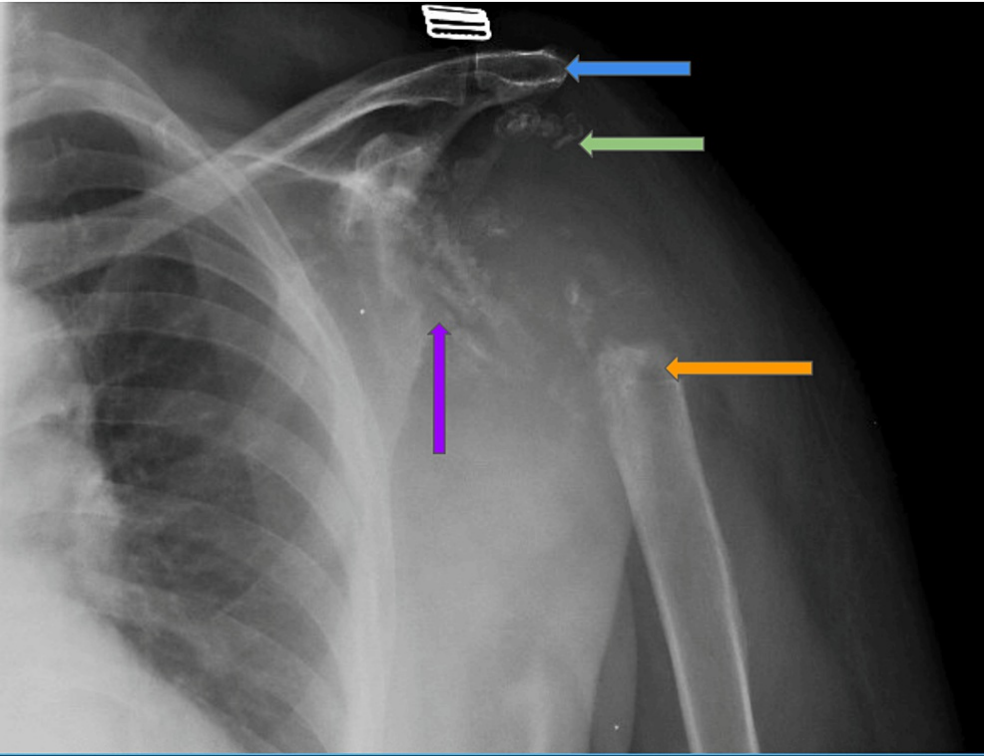
X-ray of the left shoulder. Displayed significant bone destruction involving the proximal humerus, humeral head (green arrow), proximal humeral shaft (orange arrow), glenoid process (purple arrow), and acromion (blue arrow).

An X-ray of the left shoulder displayed significant bone destruction, involving substantial portions of the proximal humerus, including the humeral head, proximal humeral shaft, glenoid process, and acromion (Figure 1).

**Figure 2:**
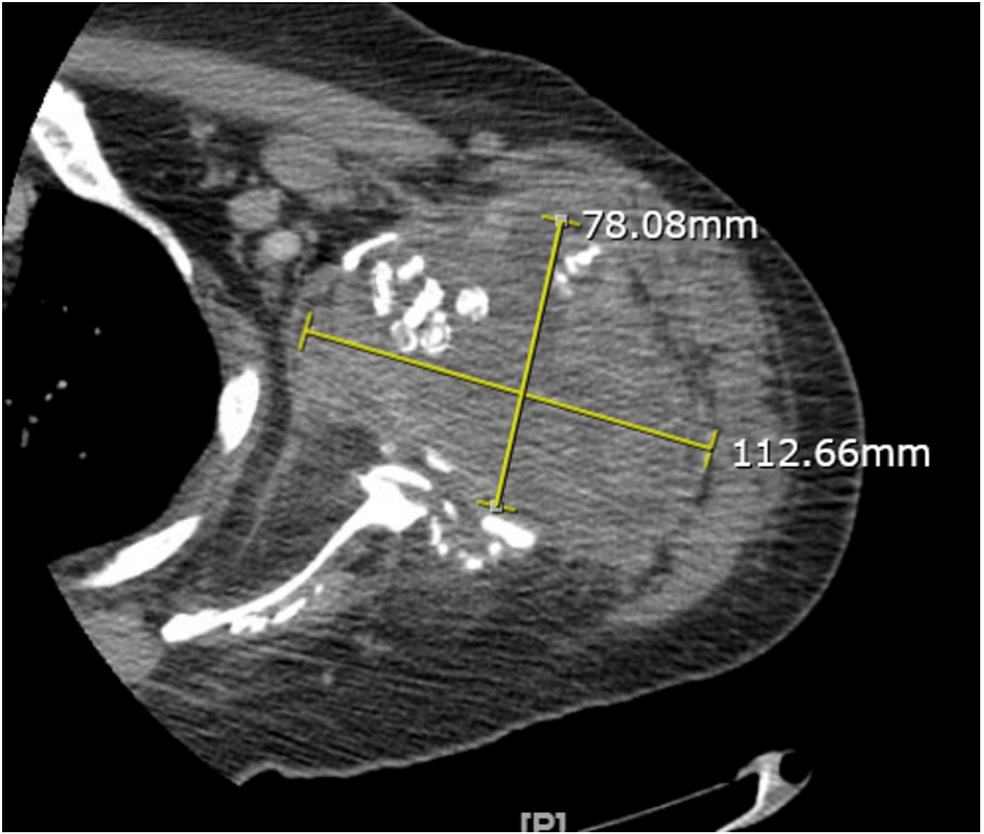
CT of the left shoulder: Large complex mass or fluid collection measuring 10 x 7.7 x 8.3 cm. A lobular mass extends posteriorly along the posterior left upper arm musculature.

Computed tomography of the left shoulder reports a large complex mass or fluid collection measuring 10 x 7.7 x 8.3 cm. There is a lobular mass extending posteriorly along the posterior left upper arm musculature, as shown in Figure 2.

**Figure 3:**
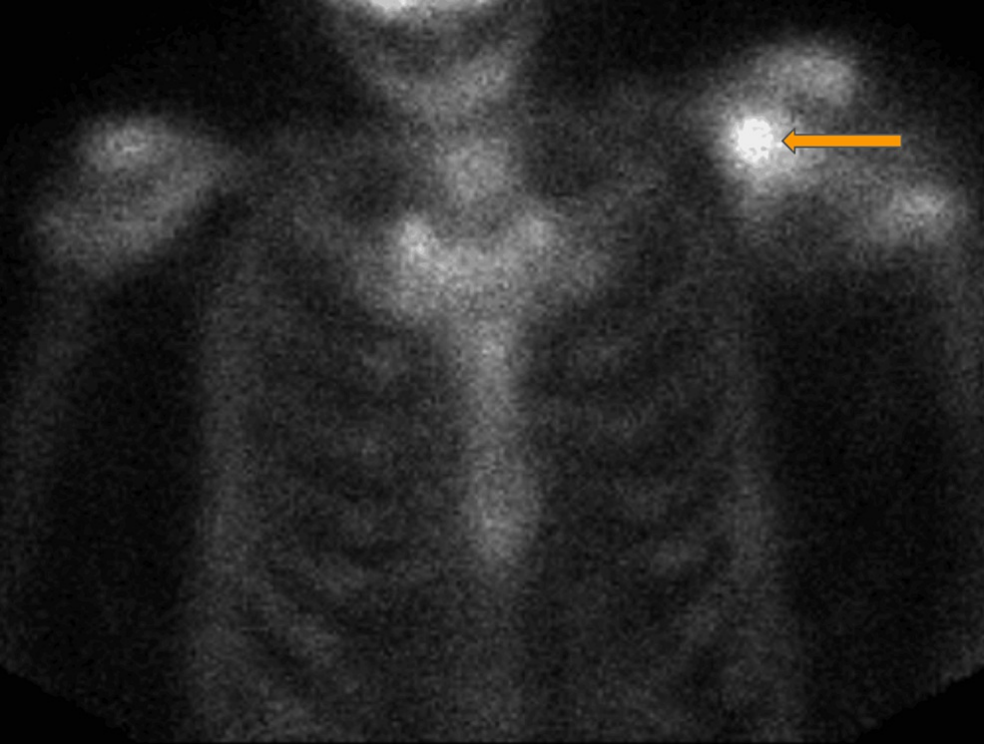
Whole body scan. Expansile osseous lesion in proximal left humerus (orange arrow).

A whole-body scan reports an expansile osseous lesion in the proximal left humerus seen primarily without radiopharmaceutical uptake, consistent with a primarily non-osseous soft tissue etiology, as shown in Figure 3.

Additional laboratory investigations were conducted to rule out other potential diagnoses. The rheumatoid factor was non-reactive, and uric acid levels were within the normal range. Urine and serum immunofixation reported IgM monoclonal protein with lambda chain specificity, and the monoclonal bands are faint, as shown in Table 1. A repeat examination one month later showed no evidence of monoclonal bands.

**Table 1:** Laboratory investigations.

Test	Initial Result	Repeat result (1 month later)
Rheumatoid Factor	Non-reactive	
Uric Acid Levels	With in normal range	
Urine and Serum Immunofixation	IgM monoclonal protein with lambda chain specificity; monoclonal bands faint	No evidence of monoclonal bands

Subsequently, a bone biopsy of the left shoulder was performed, ruling out malignancy. This biopsy was carried out at a different facility.

## Discussion

Syringomyelia, characterized by a fluid-filled cavity in the spinal cord causing compression of spinothalamic tract fibers, results in the loss of pain and temperature sensation and contributes to joint deformities [1, 2]. This condition often leads to Charcot arthropathy of the shoulder, also known as NA, a rare, progressive degenerative disease causing severe functional impairment and reduced range of motion, as seen in the presented case. Typically affecting middle-aged males and more commonly observed in weight-bearing joints like the foot and ankle, NA can also affect non-weight-bearing joints such as the shoulder and elbow. Neurological conditions like syringomyelia [3] and diabetes often underlie NA, while trauma, neurosyphilis, multiple sclerosis, osteoarthritis, rheumatoid arthritis, septic arthritis, end-stage renal disease, and chronic alcoholism are other potential causes. Symptoms of NA can manifest early as painless, swollen shoulders with limited range of motion, progressing to loss of sensation, temperature, and areflexia in later stages.

The diagnosis of NA relies on a comprehensive assessment involving clinical signs, symptoms, radiological imaging, and additional tests aimed at excluding other potential causes, including soft tissue tumors. Early X-ray findings may demonstrate degenerative changes, fragmentation, and deformities resembling osteoarthritis. In later stages, radiographic observations may include flattening of the humeral head, soft tissue calcifications, joint subluxation, and eventual dislocation. CT scans are useful in ruling out chronic infections or osteomyelitis, while MRI provides detailed information about soft tissue involvement and helps differentiate NA from other conditions. An MRI of the cervical spine can be particularly helpful in excluding a syrinx when a neuropathic shoulder is observed [2].

Bone scans can detect neuropathic joints and osteomyelitis, although they lack specificity. Indium WBC scans aid in distinguishing neuropathic joints from osteomyelitis. When the cause is unclear or when there is suspicion of an underlying malignant or infectious process, a biopsy is performed. Biopsies are vital for differentiating potential causes and planning appropriate treatment, especially in cases where NA may be a result of chronic or subacute infections, or when an underlying malignancy such as osteosarcoma mimics the presentation of NA.

**Figure 4:**
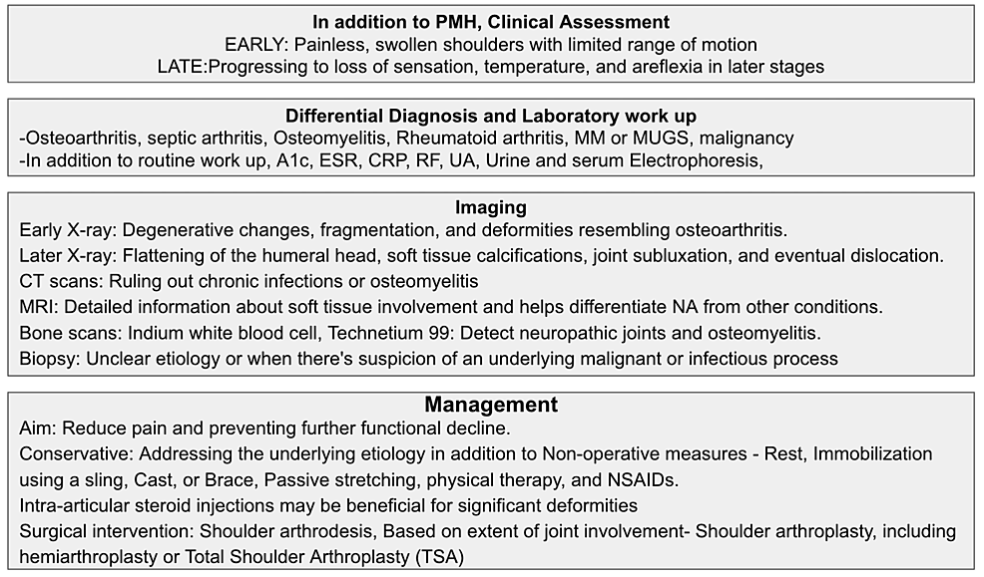
Summary of neuropathic arthropathy of the shoulder joint. MM: Multiple Myeloma; MGUS: Monoclonal Gammopathy of Undetermined Significance; ESR: Erythrocyte Sedimentation Rate; CRP: C-reactive Protein; RF: Rheumatoid Factor; UA: Uric Acid; NA: Neuropathic Arthropathy.

NA affecting the shoulder is a complex condition, and its management is tailored to the unique circumstances of each individual, with the aim of reducing pain and preventing further functional decline. Conservative approaches involve non-operative measures such as rest, immobilization using a sling, cast, or brace, passive stretching, physical therapy, and NSAIDs. The primary objective of conservative management is to halt the progression of pain and swelling while preserving joint function. In cases where syringomyelia is the underlying cause, neurosurgical decompression of the syrinx is advised [4]. Studies have indicated regrowth of the glenoid fossa following syrinx decompression. Approximately 50% of patients have reported improvement with non-operative management. For those with significant deformities, intra-articular steroid injections may be beneficial, and surgical intervention may be necessary to restore joint function if conservative management proves ineffective. Initially, surgical intervention for NA involving the shoulder joints often entailed shoulder arthrodesis. However, current studies suggest that the extent of joint involvement determines the suitability of options such as shoulder arthroplasty, including hemiarthroplasty or Total Shoulder Arthroplasty (TSA), which has shown enhanced functional outcomes. Summary of NA of shoulder joint depicted in Figure 4. 

Prognosis is influenced by the underlying cause, disease stage, and response to therapy, emphasizing the importance of early diagnosis and appropriate intervention for improved outcomes [5].

## Conclusions

Given the infrequency of NA in the shoulder, the available literature predominantly comprises case series and reports. Detailed patient cases aid in distinguishing NA from other conditions, shaping treatment strategies, and ultimately enhancing outcomes. This case study, in particular, illuminates the challenges of diagnosing and managing NA associated with cervical and thoracic syringomyelia, underscoring the need for a comprehensive, multidisciplinary approach to address these complex conditions.
